# Guidelines for Data Acquisition, Quality and Curation for Observational Research Designs (DAQCORD)

**DOI:** 10.1017/cts.2020.24

**Published:** 2020-03-13

**Authors:** Ari Ercole, Vibeke Brinck, Pradeep George, Ramona Hicks, Jilske Huijben, Michael Jarrett, Mary Vassar, Lindsay Wilson, the DAQCORD collaborators

**Affiliations:** 1 Department of Medicine, Division of Anaesthesia, University of Cambridge, Cambridge, UK; 2 QuesGen Systems, Inc, Burlingame, CA, USA; 3 International Neuroinformatics Coordinating Facility, Karolinska Institutet, Stockholm, Sweden; 4 One Mind, Rutherford, CA, USA; 5 Department of Public Health, Center for Medical Decision Sciences, Erasmus MC, Rotterdam, The Netherlands; 6 Department of Neurological Surgery, University of California, San Francisco, CA, USA; 7 Division of Psychology, University of Stirling, Stirling, UK

**Keywords:** Data quality, curation, observational studies, Delphi process, design, reporting

## Abstract

**Background:**

High-quality data are critical to the entire scientific enterprise, yet the complexity and effort involved in data curation are vastly under-appreciated. This is especially true for large observational, clinical studies because of the amount of multimodal data that is captured and the opportunity for addressing numerous research questions through analysis, either alone or in combination with other data sets. However, a lack of details concerning data curation methods can result in unresolved questions about the robustness of the data, its utility for addressing specific research questions or hypotheses and how to interpret the results. We aimed to develop a framework for the design, documentation and reporting of data curation methods in order to advance the scientific rigour, reproducibility and analysis of the data.

**Methods:**

Forty-six experts participated in a modified Delphi process to reach consensus on indicators of data curation that could be used in the design and reporting of studies.

**Results:**

We identified 46 indicators that are applicable to the design, training/testing, run time and post-collection phases of studies.

**Conclusion:**

The Data Acquisition, Quality and Curation for Observational Research Designs (DAQCORD) Guidelines are the first comprehensive set of data quality indicators for large observational studies. They were developed around the needs of neuroscience projects, but we believe they are relevant and generalisable, in whole or in part, to other fields of health research, and also to smaller observational studies and preclinical research. The DAQCORD Guidelines provide a framework for achieving high-quality data; a cornerstone of health research.

## Introduction

Observational studies are a crucial part of the biomedical research armamentarium, particularly when studying complex conditions or the related problem of understanding the outcomes of interventions in highly heterogeneous real-world populations [1]. As well as generalisability, the cost-benefit ratio of enrolling a subject in observational studies is relatively low, which makes feasible the recruitment of large samples potentially needed to reliably identify modest, but clinically important differences. This scalability, alongside the availability of electronic case-report form (eCRF) platforms and increasing availability of routinely collected data in electronic form, means that it is possible to devise large, multicentre/multinational observational projects.

With open or shared access to data becoming increasingly common, including with funding agencies, it is likely that large observational data sets will become important resources for future secondary analysis by external investigators. For example, a recent comparative effectiveness study in traumatic brain injury [2] was designed to prospectively acquire demographic, longitudinal clinical intervention, outcome, biomarker, ‘omics, imaging and waveform data in 5400 patients in 3 strata from multiple sites in 22 countries. This data set alone comprises more than 2500 discrete data concepts, but in addition, it is designed to be compatible with data from sister studies in the USA, Australia, India and China. This combination of scale, structure and data types makes such initiatives highly complex, technical challenges.

Even electronically collected clinical data may comprise a diverse mixture of data types and sources, and combinations of single, repeated measures, as well as time series, which may be irregularly sampled. Combining this with ‘omics, waveform recordings or imaging data introduces yet another tier of structural complexity. The involvement of multiple sites, particularly where these are international, may introduce further data variances due to local interpretation of procedures and linguistic and cultural misunderstandings. Notwithstanding incomplete data standards, real-world data from even a well-conducted study will inevitably contain errors or limitations that can only be understood in the context of the precise study structure. An understanding of this is crucial to making robust inferences and therefore also to repeatability. Furthermore, without detailed metadata, this knowledge can reside only with the study team, limiting transparency and making secondary analysis potentially subject to bias or other misinterpretations.

Data curation is clearly important, but the complexity and effort involved are under-appreciated and this may have serious scientific repercussions on the entire data sharing/open science enterprise. Poor attention to detail from design through execution including quality control and curation may severely limit data interpretation and consequently reuse and transparency. For prospective studies, post factum curation may improve data usability but retrospective correction of issues that emerge during the collection period is at best time-consuming and may be impossible. Thus, data quality efforts should start at the study design phase. Even the timely detection of emergent data quality issues is predicated on an understanding of both the data structure and study structure and will be severely hampered if these are not carefully specified.

Since a lack of attention to data quality and curation throughout the study may not only degrade data quality but also limit the validity of primary and subsequent analyses, an appraisal of this is important in evaluating study quality. Initiatives such as the Strengthening the Reporting of Observational studies in Epidemiology (STROBE) guidelines [3] aim to improve transparency and reproducibility in observational research. However, STROBE primarily addresses crucial conceptual and statistical rigour. A more recent extension to STROBE, the REporting of studies Conducted using Observational Routinely-collected Data (RECORD) checklist [4], touches on data quality in the context of routine data. However, neither of these excellent initiatives directly address the equally critical question of the extent or adequacy of the steps taken to ensure the data are high quality, or to more fully inform a reader of any potential limitations to the analysis resulting from the curation process. This also means that study designers lack a prospective framework from which to devise (and budget) the necessary comprehensive data quality strategy at study conception and design.

The Data Acquisition, Quality and Curation for Observational Research Designs (DAQCORD) Guidelines were developed for investigators conducting large observational research studies to aid the design, documentation and reporting of practices for assuring data quality within their studies. This information is intended to provide guidance and a transparent reporting framework for improving data quality and data sharing. Given the absence of a structured framework for the description and appraisal of the collection and curation process, the DAQCORD Collaboration aims to address these issues and has three key aims.

1. To provide a framework/toolkit for robust study design (and eCRF design in particular) and data quality management.
2. To provide a framework by which proposed study plans can be systematically appraised (for example, by funding organisations) in terms of their approach to data quality.
3. To provide a reporting framework with which to describe the steps taken to ensure data quality in the final study publication.

## Methods

### Development of the DAQCORD Indicators

**Fig. 1.**
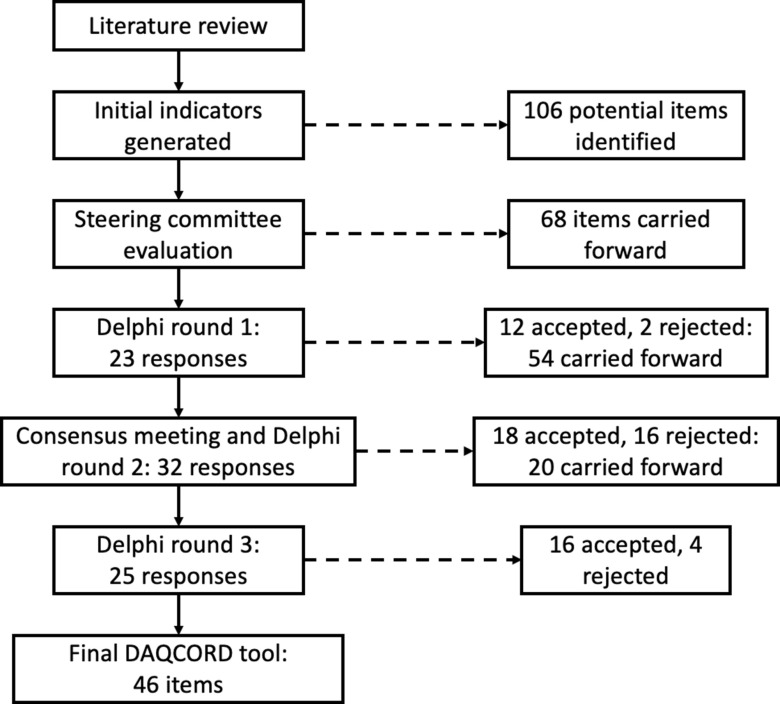
Flow diagram for the DAQCORD-modified Delphi process.

The DAQCORD project was initiated in 2017, originally arising from discussion of data management issues in the InTBIR [5] consortium. This consortium includes observational studies which are representative of the most ambitious staged to date in the field of traumatic brain injury with respect to the number of patients and complexity of the data collected. Funding/technical support was obtained to facilitate a face-to-face consensus meeting as well as the necessary survey infrastructure and website (www.daqcord.org). Our methodology was designed in accordance with best practice published by the Equator network [6] with which the initiative was registered. We formed a Steering Committee consisting of seven individuals with professional backgrounds in informatics and data management and/or experience in data curation/data set design in large-scale observational studies. A summary of the steps involved in developing the DAQCORD indicators is shown in Figure 1.

The Steering Committee performed a search of literature for relevant publications on data quality methodology for large observational and heterogeneous studies. Sources consulted included PubMed, Ovid-Medline, Web of Science and Google Scholar, and we followed this up by hand searching specific journals. The search identified a range of informing literature, including a body of work concerning data collected during routine care [7–10]; however, we were unable to identify any peer-reviewed publications giving systematic practical advice on data quality methodology for observational studies (i.e. studies with a typical cycle of design, implementation and post-collection). The Steering Committee generated an initial set of 106 items potentially relevant to data quality that were derived from published sources, including transferable concepts identified by the Steering Committee from our literature search [11–17], unpublished manuals on data curation provided by studies within the InTBIR consortium, previously published Equator guidelines, and from personal experience. We carried out an initial exercise within the Steering Committee to categorise questions on the data quality factors of completeness, correctness, concordance, plausibility and currency (Weiskopf and Weng [7], see Table 1 for definitions of these terms) and evaluate the importance of individual items. Items were reviewed for duplication and overlap and were removed or re-written as necessary. As a result of this initial exercise, the number of items was reduced to 68 and the remaining items were edited for clarity.

**Table 1.** Key terms and concepts

Terms	Definition in the context of use for the DAQCORD Guidelines
Action ability	The indicator can be acted upon in the data curation process to assure quality.
Completeness	The degree to which the data were in actuality collected in comparison to what was expected to be collected.
Concordance	The agreement between variables that measure related factors.
Correctness	The accuracy of the data and its presentation in a standard and unambiguous manner.
Currency	The timeliness of the data collection and representativeness of a particular time point.
Curation	The management of data throughout its lifecycle (acquisition to archiving) to enable reliable reuse and retrieval for future research purposes.
Data	Information that is collected and stored electronically for primary and secondary analysis of health-related research.
Data quality factors	The completeness, correctness, concordance, plausibility and currency of data
Feasibility	Information about the indicator is available or easy to obtain.
Indicator	A measurable variable that is used to represent the quality of the data curation methods.
Observational study	Any research study involving data collection without a manipulation or intervention
Plausibility	The extent to which data are consistent with general medical knowledge or background information and are therefore believable
Validity	The indicator reflects the quality of the data curation methods used in the research.

The Steering Committee agreed a Delphi approach to reach consensus on the DAQCORD tool was appropriate, with the modification of having a face-to-face meeting of the panel in addition to circulation of material. A meeting was judged vital to allow in-depth discussion of the aims and outcomes of the project as well as the criteria and boundaries applied to item selection. The 68 items were collated into an online structured questionnaire for rating by panel members, and a consensus conference was held in September 2018 at the National Institutes of Health, Bethesda. There was a range of expertise among the 46 panel participants, including 9 bioinformaticists/computer scientists, 8 data managers/data scientists, 7 epidemiologists/statisticians, 15 clinician/researchers and 7 biomedical scientists. The majority were from the USA (29), with 9 from Europe and 8 from Canada. Participants also represented a range of organisations, including 33 from academia, 8 from government, 3 from non-profit organisations and 2 from industry. Respondents were chosen to be representative of a range of career stages from principal investigators to earlier stage researchers.

At the consensus meeting, we discussed the criteria used to assess the suitability of items for assessing data quality; the criteria agreed were validity, feasibility and action ability. The three criteria were elaborated as follows: “validity” means that “the metric is likely to reflect data quality”, “feasibility” means “this is something that can be measured or assessed and is quantifiable”, and “action ability” means that “improving this metric could be used in practice to make changes to a study that improves data quality”. We also discussed whether additional items were required, the potential applications of the instrument, and strategies for disseminating the outputs of the project. The consensus meeting allowed greater convergence on key issues and more detailed feedback on responses than would have been possible using only online questionnaires.

In the separate rounds of the Delphi, panel members rated items on whether they met each criterion using a Likert-type scale from 1 (strongly disagree) to 5 (strongly agree). A formal procedure was agreed for adopting and rejecting items on the basis of ratings which was in keeping with methods which have been previously employed and found to provide consensus [18,19]. A median score ≥ 4 for agreement was considered a good rating for the dimension, while ≤3 was a neutral or poor rating. In addition, an interquartile range of 0 or 1 was regarded as very good consensus on the rating, 2 as good consensus and more than 2 as a lack of consensus. To be accepted, an item needed a good rating on each dimension and a good consensus on each rating (or very good consensus for the “validity” dimension), items were rejected if they had a low rating on one or more dimensions with good consensus and otherwise they were carried forward to the next round. The criteria adopted for “validity” ratings were stricter because this dimension was regarded as critical to the usefulness of the item. No upper or lower boundary was set on the number of items that would be accepted. Respondents could also make free text comments, which were included in the feedback to participants. At each stage, items were also edited for precision or duplication as a result of responses from participants. Respondents were able to see results for each item in each domain from previous rounds.

## Results

The Delphi process converged on 46 items after 3 rounds that were judged to be indicators of data quality (see Figure 1). The 46 items (henceforth referred to as indicators) included in the final set all had median ratings for validity, feasibility and action ability of 4 or 5 indicating agreement or strong agreement that the component met the criterion. All the indicators also showed good consensus after three rounds. The final DAQCORD components are categorised and listed by data quality factors (i.e. completeness, correctness, concordance, plausibility and currency) with the relevant study phase for implementation noted in a separate column (see Table 2). Supplementary material, including the DAQCORD indicators with examples derived from the Delphi exercise, is also presented online (https://www.daqcord.org/daqcord-questions/).

**Table 2.** DACQORD indicators

Study phase	Dimension	Indicator
Design time	Correctness	The case report form (CRF) has been designed by a team with a range of expertise.
Design time	Completeness	There is a robust process for choosing and designing the data set to be collected that involves appropriate stakeholders, including a data curation team with appropriate skill mix.
Design time	Concordance	The data ontology is consistent with published standards (common data elements) to the greatest extent possible.
Design time	Concordance	Data types are specified for each variable.
Design time	Correctness	Variables are named and encoded in a way that is easy to understand.
Design time	Representation	Relational databases have been appropriately normalised: steps have been taken to eliminate redundant data and remove potentially inconsistent or overly complex data dependencies.
Design time	Representation	Each individual has a unique identifier.
Design time	Representation	There is no duplication in the data set: data have not been entered twice for the same participant.
Design time	Completeness	Data that are mandatory for the study are enforced by rules at data entry, and user reasons for overriding the error checks (queries) are documented in the database.
Design time	Completeness	Missingness is defined and is distinguished from “not available”, “not applicable”, “not collected” or “unknown”. For optional data, “not entered” is differentiated from “not clinically available” depending on research context.
Design time	Plausibility	Range and logic checks are in place for CRF response fields that require free entry of numeric values. Permissible values and units of measurement are specified at data entry.
Design time	Correctness	Free text avoided unless clear scientific justification and (e.g. qualitative) analysis plan specified and feasible.
Design time	Concordance	Database rule checks are in place to identify conflicts in data entries for related or dependent data collected in different CRFs or sources.
Design time	Representation	There are mechanisms in place to enforce/ensure that time-sensitive data are entered within allotted time windows.
Design time	Completeness	There is clear documentation of interdependence of CRF fields, including data entry skip logic.
Design time	Correctness	Data collection includes fields for documenting that participants meet inclusion/exclusion criteria.
Design time	Representation	The data entry tool does not perform rounding or truncation of entries that might result in precision loss.
Design time	Plausibility	Extract/transform/load software for batch upload of data from other sources such as assay results should flag impossible and implausible values.
Design time	Representation	Internationalisation is undertaken in a robust manner, and translation and cultural adaption of concepts (e.g. assessment tools) follow best practice.
Design time	Concordance	Data collection methods are documented in study manuals that are sufficiently detailed to ensure the same procedures are followed each time.
Design time	Correctness	All personnel responsible for entering data receive training and testing on how to complete the CRF.
Design time	Correctness	The CRF/eCRF is easy to use and include a detailed description of the data collection guidelines and how to complete each field in the form. They are pilot-tested in a rigorous pre-specified and documented process until reliability and validity are demonstrated.
Design time	Concordance	Data collectors are tested and provided with feedback regarding the accuracy of their performance across all relevant study domains.
Design time	Correctness	Data collection that requires specific content expertise is carried out by trained and/or certified investigators.
Design time	Correctness	Assessors are blinded to treatment allocation or predictor variables where appropriate and such blinding is explicitly recorded.
Design time	Correctness	There is a clear audit chain for any data processing that takes place after entry, and this should have a mechanism for version control if it changes.
Design time	Representation	Data are provided in a form that is unambiguous to researchers.
Design time	Concordance	For physiological data, the methods of measurement and units are defined for all sites.
Design time	Correctness	Imaging acquisition techniques are standardised (e.g. magnetic resonance imaging).
Design time	Correctness	Biospecimen preparation techniques are standardised.
Design time	Correctness	Biospecimen assay accuracy, precision, repeatability, detection limits, quantitation limits, linearity and range are defined. Normal ranges are determined for each assay.
Design time	Correctness	There is automated entry of the results of biospecimen samples.
Training and testing	Completeness	A team of data curation experts are involved with pre-specified initial and ongoing testing for quality assurance.
Run time	Completeness	Proxy responses for factual questions (such as employment status) are allowed in order to maximise completeness.
Run time	Representation	Automated variable transformations are documented and tested before implementation and if modified.
Run time	Completeness	There is centralised monitoring of the completeness and consistency of information during data collection.
Run time	Plausibility	Individual data elements should be checked for missingness. This should be done against pre-specified skip-logic/missingness masks. This should be performed throughout the study data acquisition period to give accurate “real time” feedback on completion status.
Run time	Plausibility	Systematic and timely measures are in place to assure ongoing data accuracy.
Run time	Correctness	Source data validation procedures are in place to check for agreement between the original data and the information recorded in the database.
Run time	Plausibility	Reliability checks have been performed on variables that are critical to research hypotheses, to ensure that information from multiple sources is consistent.
Run time	Correctness	Scoring of tests is checked. Scoring is performed automatically where possible.
Run time	Correctness	Data irregularities are reported back to data collectors in a systematic and timely process. There is a standard operating procedure for data irregularities to be reported back to the data collectors and for documentation of the resolution of the issue.
Run time	Representation	Known/emergent issues with the data dictionary are documented and reported in an accessible manner.
Post-collection	Representation	The version lock-down of the database for data entry is clearly specified.
Post-collection	Correctness	A plan for ongoing curation and version control is specified.
Post-collection	Representation	A comprehensive data dictionary is available for end users.

The DAQCORD indicators are intended as a descriptive system for planning and reporting observational studies. At a minimum, they can be used as a checklist for documenting whether an indicator is being addressed fully, partially or not at all. A more extended and informative record can be made by users through creation of a brief narrative for each indicator describing how this was addressed for their study. The resulting text will provide formal documentation of the data quality steps taken for the study, which will serve as an evidential record that can inform funders and the research community.

## Discussion

The DAQCORD Guidelines were developed to help authors in reporting on large observational studies and to assist readers and reviewers in appraising data quality in published studies and of the data set as a whole. Furthermore, the Guidelines aim to provide a prospective framework to encourage comprehensive best practice in the design of a data quality strategy from the outset to ensure that the data ultimately collected is of as high quality as possible, to streamline and limit the need for costly retrospective curation, as well as to improve transparency and facilitate meaningful open access and reuse. It may also provide a structure for funding agency review of proposed data quality strategies.

DAQCORD was developed by a panel selected for its comprehensive expertise in the practical design and issues encountered in large data-heavy observational studies. It is likely that observational data sets will grow in complexity and scope in the future, and it is conceivable that new challenges (or indeed data platforms and standards) will emerge and consequently DAQCORD will need to be revised in the light of such developments.

Observational studies are, by their nature, heterogeneous in their domains; aims and scope and therefore not all elements will be relevant to all study designs. At the same time, we believe that where they are applicable, the indicators that we have developed provide a systematic framework for addressing potential data quality issues. It is not our aim to prescriptively specify the steps necessary for all studies. Indeed, given the heterogeneity of such studies, we do not believe that this is possible. There may be many, equally valid, ways in which a particular study may address (or demonstrate that it has addressed) any particular aspect of data curation. As part of the Delphi process, we also gathered examples of possible best practice for each indicator: these are available online to serve as a guide and further elaboration. We also envision this a “living resource”, which could be expanded on to include more indicators for selected types of data, i.e., electronic health records, preclinical research, qualitative data (e.g. derived from interviews and surveys), neuroimaging, biospecimens, continuous physiological measurements, etc.

The indicators are weighted towards measures that should be implemented at design time. In our experience, the challenges presented by large-scale projects may be under-appreciated at project inception. In particular, the amount of funding that needs to be allocated to data quality processes may be underestimated. Grant giving bodies could play a key role in identifying this shortfall at proposal stage and ensuring that it is adequately addressed.

We recognise that there are likely to be limitations to the retrospective application of the Guidelines to existing data sets. For some studies, the details of the steps taken during data curation may not be available. It may also be appropriate to be tolerant when applying criteria post hoc, since the original study may not have had the resources to adequately address data curation at the time. Issues in such databases may be addressed over time, for example, through documentation of known problems by researchers.

DAQCORD set out to address the issues of large-scale, complex observational studies, explicitly including the design of the data capture infrastructure such as eCRFs since this is an area which is highly complex and potentially problematic. A large proportion of the Delphi collaborators are from neurosciences backgrounds. This domain has seen some of the most complex data sets from large-scale multinational observational studies, and therefore, this community has necessarily developed a substantial expertise in this area. However, we believe that the concepts are generalisable to other clinical disorders, and smaller clinical and preclinical studies, as well. In summary, we believe that the DAQCORD Guidelines will enhance the design and management of biomedical research studies, provide assurance to potential collaborators about data quality and promote collaborative research to improve healthcare on a global scale.

## Supplementary material

For supplementary material accompanying this paper visit https://doi.org/10.1017/cts.2020.24. click here to view supplementary material
