# Supplementary material for: Guidelines for Data Acquisition, Quality and Curation for Observational Research Designs (DAQCORD)
Source: J Clin Transl Sci. 2020 Mar 13;4(4):354–9. doi: 10.1017/cts.2020.24 (PMC7681114; doi:10.1017/cts.2020.24)
Supplement: Supplementary file 1 [file S2059866120000242sup001.docx]

**DAQCORD Questions**

| **Study Phase** | **Dimension** | **Question** |
| --- | --- | --- |
| Design-time | Correctness | 1. The case report form (CRF) has been designed by a team with a range of expertise. *Examples: 1: CRF design teams were designated and followed a defined process for end-user acceptance testing to ensure it aligns with the workflow and research milestones. 2: Design and testing included experts in areas under study such as database design and curation; statistics, neuroimaging; biospecimen management; outcome assessments; and clinical assessments etc. 3: Before data entry was started, the CRF forms were reviewed by an independent researcher with appropriate expertise.* |
| Design-time | Completeness | 2. There is a robust process for choosing and designing the dataset to be collected that involves appropriate stakeholders, including a data-curation team with appropriate skill mix. *Example: 1: Data governance team should include all the important and key stakeholders such as: data curator, steward, analysts. 2: Subject matter experts should be involved from the outset (study design time) to ensure that the data is sufficient and aligned to fullfill the study objectives. 3: Technical experts should be involved from the outset (study design time) to ensure that the variables to be collected are appropriately specified.* |
| Design-time | Concordance | 3. The data ontology is consistent with published standards (common data elements) to the greatest extent possible. *Examples: 1: Data base fields conform as appropriate to FITBIR NINDS Common Data Elements for Traumatic Brain Injury; CDISC; CDASH; and Abbreviated Injury Scores. 2: Pre-specified data ontology at design-time. 3: Published data elements used as far as possible. However, when not possible, deviations from published specifications clearly documented and have robust scientific justification.* |
| Design-time | Concordance | 4. Data-types are specified for each variable. *Example: 1: Specifications for floating point, integer, factor or free text* |
| Design-time | Correctness | 5. Variables are named and encoded in a way that is easy to understand. *Examples: 1: When data is extracted from the database, it should be easy to recognize what the variable names refer to. 2: When there are many variables of the same kind, the names follow a pattern that is consistent. 3: The same name should be used across the CRF and database design so that confusion is minimised.* |
| Design-time | Representation | 6. Relational databases have been appropriately normalised: steps have been taken to eliminate redundant data and remove potentially inconsistent or overly complex data dependencies. *Example: 1: Similar data will be collected in one table. 2: Repeating data is not collected in separate fields, but rather identified by a time point indicator.* |
| Design-time | Representation | 7. Each individual has a unique identifier. *Example: 1: The GUID (Global Unique IDentifier) or the GUPI (Generated Unique Patient Identifier) is a method of ensuring that patients are not duplicated across datasets.* |
| Design-time | Representation | 8. There is no duplication in the data set: data has not been entered twice for the same participant. *Examples: 1: Duplication identification process in place to assure single entry of codes assigned to unique subjects by institution across repository data sets (e.g., blood samples, imaging sequences and other assessments). 2: Audits are conducted to reconcile record linkages and assure no duplicates are entered (e.g., image sequence files not submitted twice; subject CRF only entered once in database; or biospecimen have unique IDs at different timepoints and only entered once). 3: For data across multiple platforms assure same subject ID is used in the database. For example, Global unique identifier (GUID).* |
| Design-time | Completeness | 9. Data that is mandatory for the study is enforced by rules at data entry and user reasons for overriding the error checks (queries) are documented in the database. *Examples: 1: Mandatory elements require a value or explanation for reason missing. 2: Curation team is responsible for reviewing and accepting or rejecting explanations. 3: Data completeness for key variables is checked against pre-specified study design goals and minimum standards for data completeness in key areas are met. 4: Quality control is in place to ensure that completed clinical measurements or investigations such as imaging meet the specifications in the study protocol.* |
| Design-time | Completeness | 10. Missingness is defined and is distinguished from ‘not available’, ‘not applicable’, ‘not collected’ or ‘unknown.’ For optional data, ‘not entered’ is differentiated from ‘not clinically available’ depending on research context. *Examples: 1: Definitions are agreed upon at design. 2: Codes are defined as appropriate to study settings: missing data from hospital record (e.g. not recorded) versus missing data from a study appointment (e.g. subject did not return etc.).* |
| Design-time | Plausibility | 11. Range and logic checks are in place for CRF response fields that require free entry of numeric values. Permissible values and units of measurement are specified at data entry. *Examples: 1: Avoid free text fields when possible. 2: eCRF has automated error flags to prompt immediate alert to review entries that don’t pass validation with option to override when appropriate.* |
| Design-time | Correctness | 12. Free text avoided unless clear scientific justification and (e.g. qualitative) analysis plan specified and feasible. *Examples: 1: ‘Other’ checkbox included in field response options as appropriate with free field text for description. 2: Free-text fields set as containing PHI to avoid inadvertent export/release of personal information.* |
| Design-time | Concordance | 13. Database rule checks are in place to identify conflicts in data entries for related or dependent data collected in different CRFs or sources. *Examples: 1: Scheduled queries are run for data checks across tables/CRFs for variables prone to conflict. 2: There should be rules to ensure that incompatible choices are excluded both within a data element (e.g. cannot be both male and female) and between related elements (e.g. male cannot be pregnant).* |
| Design-time | Representation | 14. There are mechanisms in place to enforce / ensure that time-sensitive data is entered within allotted time windows. *Examples: 1: Database has flags for identification of when CRF completion or a study visit is overdue. 2: Automated weekly messages sent to coordinators listing overdue eCRFs. 3: Central study staff responsible for monitoring overdue CRFs.* |
| Design-time | Completeness | 15. There is clear documentation of interdependence of CRF fields, including data entry skip logic. *Examples: 1: For data entry: cells for skipped questions are closed. 2: Clear directions on CRFs for when to skip questions.* |
| Design-time | Correctness | 16. Data collection includes fields for documenting that participants meet inclusion/ exclusion criteria. *Examples: 1: Avoid enrollment errors by requiring completion of a CRF with checklist confirming that eligibility criteria have been met for enrolled subjects. 2: For subjects that need to be enrolled within specific window of time there are timestamp checks in the eCRF in place to verify the eligibility window. 3: Date of birth falls within the enrollment age criteria.* |
| Design-time | Representation | 17. The data entry tool does not perform rounding or truncation of entries that might result in precision-loss. *Example: 1: Where decimal places are to be limited, it should be clear to the user and prevent them from entering the value rather than a truncation performed with the user being unaware.* |
| Design-time | Plausibility | 18. Extract / transform / load software for batch upload of data from other sources such as assay results should flag impossible and implausible values. *Examples: 1: ETL process logged. 2: Permissible value ranges in the ETL software are study specific rather than relying on institution permissible value logic.* |
| Design-time | Representation | 19. Internationalisation is undertaken in a robust manner, and translation and cultural adaption of concepts (e.g. assessment tools) follows best practice. *Examples: 1: Systematic approach to translation and cross-cultural validation of study material in multi-language settings. 2: Psychometric transformations considered.* |
| Design-time | Concordance | 20. Data collection methods are documented in study manuals that are sufficiently detailed to ensure the same procedures are followed each time. *Examples: 1: Manuals compiled by multidisciplinary team of stakeholders and involve data curation expertise. For example, Imaging studies and biospecimen collection procedures are detailed for study personnel and end-users. 2: Manuals specify how eCRF data is collected, when, by what metrics and under what conditions. 3: CRF fields have links to relevant data manual sections.* |
| Design-time | Correctness | 21. All personnel responsible for entering data receive training and testing on how to complete the CRF. *Examples: 1: Training materials developed and disseminated via print, webinar or in-person training. 2: Study implementation checklist includes documentation for training requirements according to study personnel roles. 3: Testing process in place: written exams with scenarios for coding data; video demonstrations for examiner competency to administer of outcome assessments; competency for performing research imaging sequences; or testing with mock patient records for complex forms. 4: Post-launch procedures for assuring new project personnel also fulfill training requirements.* |
| Design-time | Correctness | 22. The CRF / eCRF are easy to use and include a detailed description of the data collection guidelines and how to complete each field in the form. They are pilot tested in a rigorous pre-specified and documented process until reliability and validity are demonstrated. *Examples: 1: Layout of paper and eCRFs are easy to navigate with simple wording. 2: Examples are provided for coding of complex response choices. 3: The CRF is designed and validated to be unambiguous to different users. 4: eCRF forms are designed to be presented based on rules to appear at times that are appropriate depending on the study design: Hiding irrelevant forms and to function as a prompt to promote timely data collection.* |
| Design-time | Concordance | 23. Data collectors are tested and provided with feedback regarding the accuracy of their performance across all relevant study domains. *Examples: 1: Random audits of clinical source documents against data entered on hard copy and eCRFs. 2: Double data entry audits of selected forms that are known to be prone to errors. 3: Audit of eCRF and hard copy logs for biospecimens; audit of complex assessments by experts. 4: Training and on-line documentation of how to handle exceptions.* |
| Design-time | Correctness | 24. Data collection that requires specific content expertise is carried out by trained and/or certified investigators. *Example: 1: Certification processes specified in protocols.* |
| Design-time | Correctness | 25. Assessors are blinded to treatment allocation or predictor variables where appropriate and such blinding is explicitly recorded. *Example: 1: Therapists administering the Functional Independence Measure (FIM) tool do not know who was given an experimental treatment or a placebo.* |
| Design-time | Correctness | 26. There is a clear audit chain for any data processing that takes place after entry, and this should have a mechanism for version control if it changes. *Examples: 1: Change logs maintained and audited. 2: Workflows and ETL (extract / transform / load) processes are described.* |
| Design-time | Representation | 27. Data are provided in a form that is unambiguous to researchers. *Example: 1: Table and study form fields relationships are clearly identified in the data dictionary.* |
| Design-time | Concordance | 28. For physiological data the methods of measurement and units are defined for all sites. *Examples: 1: Uniform collection standards are in place. 2: When uniform measures are not used by hospital units or labs, the CRF provides conversion guidance. 3: Inter-site sampling differences are described in the data dictionary.* |
| Design-time | Correctness | 29. Imaging acquisition techniques are standardised (e.g. magnetic resonance imaging). *Examples: 1: Phantom standardization of MRI scanners across study sites. 2: Appropriate assurance of quality and comparability between sites and within sites and assurance of ongoing compliance over time. 3: SOP aligned with agreed imaging acquisition standards. 4: Adequate training of technical personnel across sites.* |
| Design-time | Correctness | 30. Biospecimen preparation techniques are standardised. *Examples: 1: Clear Biospecimen Standard Operating Procedure (SOP) for sample preparation. 2: Lab SOP aligned with agreed sample handling standards. 3: Adequate training of lab personnel across sites. 4: Published / national / international standards should be specified and adhered to where possible. 5: Audit trail of sample processing and storage. 6: Protocol deviations filed when protocol not followed. 7: Audits of time intervals for drawing, processing and freezing are within protocol windows for each specimen type.* |
| Design-time | Correctness | 31. Biospecimen assay accuracy, precision, repeatability, detection limits, quantitation limits, linearity and range are defined. Normal ranges are determined for each assay. *Examples: 1: Normal ranges are determined for each assay. 2: Assays are standardised as far as possible. 3: Assay substrate are defined (e.g. ionised vs. unionised fractions, whole blood vs serum, total vs protein bound as appropriate). 4: Normal ranges should consider/be customised to pathology or subject group in question.* |
| Design-time | Correctness | 32. There is automated entry of the results of biospecimen samples *Example: 1: Vial labels IDs entered into bio-repository logs via bar code scanner.* |
| Training and Testing | Completeness | 33. A team of data-curation experts are involved with pre-specified initial and ongoing testing for quality assurance. *Examples: 1: Anticipated and unanticipated data quality issues are identified and appropriate solutions/mitigation strategies are devised in a timely manner. 2: There is a process in place for ongoing systematic quality checking and analysis of data during data acquisition to identify unexpected data quality issues in order to remedy them in a timely manner.* |
| Run-time | Completeness | 34. Proxy responses for factual questions (such as employment status) are allowed in order to maximize completeness. *Example: 1: Protocol is designed to allow for surrogate to respond to selected questions when patient is too impaired to respond. CRF indicates when surrogate is responding.* |
| Run-time | Representation | 35. Automated variable transformations are documented and tested before implementation and if modified. *Examples: 1: For example, testing is carried out if fields such as ‘years of education’ and ‘level of education’ are automatically combined into a new variable summarising educational status. 2: Coding is tested on a subset of data, ideally by multiple members of the study team. 3: There is a centralised log / version management of all transformations used.* |
| Run-time | Completeness | 36. There is centralized monitoring of the completeness and consistency of information during data collection. |
| Run-time | Plausibility | 37. Individual data elements should be checked for missingness. This should be done against pre-specified skip-logic / missingness masks. This should be performed throughout the study data acquisition period to give accurate ‘real time’ feedback on completion status. *Example: 1: Rules in place to check whether the apparently missing element should be completed or not for a particular study stratum or time point.* |
| Run-time | Plausibility | 38. Systematic and timely measures are in place to assure ongoing data accuracy. *Examples: 1: Schedule of standard reports for data quality checks. 2: Ad hoc reports for identification of new issues.* |
| Run-time | Correctness | 39. Source data validation procedures are in place to check for agreement between the original data and the information recorded in the database. *Examples: 1: Site visits for audit of subject hospital records against CRF and eCRF entries. 2: Submission of redacted hospital records for audit against eCRF entries. 3: Independent double entry of any data transferred to the database from paper form, either for full dataset or for representative subset.* |
| Run-time | Plausibility | 40. Reliability checks have been performed on variables that are critical to research hypotheses, to ensure that information from multiple sources is consistent. *Examples: 1: Database rule checks in place to identify conflicts in data entries for related or dependent data collected in different CRFs or sources. 2: Inter-rater scoring of MRIs is done independently by 3 experts on 10 cases per month.* |
| Run-time | Correctness | 41. Scoring of tests is checked. Scoring is performed automatically where possible. |
| Run-time | Correctness | 42. Data irregularities are reported back to data collectors in a systematic and timely process. There is a standard operating procedure for data irregularities to be reported back to the data collectors and for documentation of the resolution of the issue *Examples: 1: Queries in place to identify the following types of scenarios: For 9 of 10 study sites a mandatory and critical variable is only missing in 5% of the subjects. For site 10 data is found to be missing in 30% of the subjects. For a single site study, several personnel have been collecting longitudinal study data with low numbers of missing data. A pattern of increasing numbers of missing data has been identified with a newly hired data collector. 2: Variables known to be problematic are regularly monitored by curation team and delays in site resolution of issues are reported to governing committee. 3. Aggregate data are examined for inconsistencies such as lower average mortality in patients with GCS 3 than GCS 4-5.* |
| Run-time | Representation | 43. Known/emergent issues with the data dictionary are documented and reported in an accessible manner. |
| Post-collection | Representation | 44. The version lock-down of the database for data entry is clearly specified. *Example: 1: Criteria in place for closing and locking eCRFs in the database.* |
| Post-collection | Correctness | 45. A plan for ongoing curation and version control is specified. |
| Post-collection | Representation | 46. A comprehensive data dictionary is available for end users. *Examples: 1: Database platform has feature for export of CRF field definitions and schema of table relations. 2: Dictionary has annotations as needed to provide guidance on coding decisions. 3: Dictionary cites primary sources where appropriate for uniform data collection (e.g., NINDS TBI CDEs)* |
